# A systematic review of the risk factors for suicidal ideation, suicidal attempt and completed suicide among children and adolescents in sub-Saharan Africa between 1986 and 2018: protocol for a systematic review of observational studies

**DOI:** 10.1186/s13643-018-0901-8

**Published:** 2018-12-12

**Authors:** Godfrey Zari Rukundo, Elizabeth Kemigisha, Moses Ocan, Wilson Adriko, Dickens Howard Akena

**Affiliations:** 1 Department of Psychiatry, Mbarara University of Science and Technology and African Centre for Suicide Research, Mbarara, Uganda; 2 Faculty of Interdiscilinary Studies, Mbarara University of Science and Technology, Mbarara, Uganda; 3 Department of Pharmacology & Therapeutics, Makerere College of Health Sciences, Kampala, Uganda; 4 Library, Mbarara University of Science and Technology, Mbarara, Uganda; 5 Department of Psychiatry, Makerere College of Health Sciences and African Centre for Systematic, Kampala, Uganda

**Keywords:** Suicide, Suicidal attempt, Suicidal ideation, Suicidal behaviour, Adolescents

## Abstract

**Background:**

Suicide is one of the leading causes of death among children and adolescents. Most studies about the burden and risk factors for suicide have been conducted in high-income countries. However, there is a dearth in the literature about the burden and risk factors for suicide among children and adolescents in low- and middle-income countries including within Sub-Saharan Africa (SSA). There is need to summarise the available literature about the burden and risk factors for suicide among children and adolescents in SSA. In this review, we will (a) determine the overall prevalence of suicidal ideation, suicidal attempt and completed suicide among children and adolescents in SSA; (b) describe the methods (such as hanging, firearms, overdose, poisoning, drowning and burning) used for suicidal attempt, and completed suicide among children and adolescents in SSA; and (c) document the risk factors for suicidal ideation, suicidal attempt and completed suicide among children and adolescents in SSA.

**Methods:**

The review will be conducted and reported in accordance to the preferred reporting items for systematic reviews and meta-analysis (PRISMA) statement. We will include journal articles that have documented the prevalence and risk factors for suicidal ideation, suicidal attempt and completed suicide among children and adolescents aged 5–19 years in SSA. We will also include accessible grey literature about the topic. Qualitative studies will be excluded from the study since they are limited in estimating prevalence. We will search different search engines including PUBMED, EMBASE, Psych-INFO, Cochrane Library, Africa wide-information and global health using suicide, adolescents and children, SSA as the keywords. We will use a meta-analysis, should we find that there is no heterogeneity between included studies.

**Discussion:**

This protocol describes a systematic review of observational studies reporting completed suicide, suicidal ideation and suicidal attempt among children and adolescents in sub-Saharan Africa. We anticipate that once this review is complete and published, our findings will be of interest to adolescents with suicidal behaviour, their families and caregivers, clinicians and other healthcare professionals, scientists and policy makers.

**Systematic review registration:**

PROSPERO International prospective register of systematic reviews: CRD42016048610.

**Electronic supplementary material:**

The online version of this article (10.1186/s13643-018-0901-8) contains supplementary material, which is available to authorized users.

## Background

Globally, suicide is a public health concern and one of the leading causes of death among children and adolescents [1]. Most studies about the burden and risk factors for suicide have been conducted in high-income countries (HIC). However, there is a dearth in the literature about the burden and risk factors for suicide among children and adolescents in low- and middle-income countries (LMIC) including within Sub-Saharan Africa (SSA); the majority of studies about the risk factors for suicide having been conducted among adults and in specific sub-populations [2–5]. A recent literature review by Mars et al. (2014) documented the prevalence of suicide and its risk factors on the African continent, but fell short of studying children and adolescents [6, 7]. The African annual prevalence of suicide was found to be 34,000 with incidence of 3.2 per 100,000.

Suicide is the third leading cause of death among adolescents and young adults [8–10]. A number of factors including high levels of poverty, growing unemployment, disruptions in traditional family life and the unrecognised and poorly treated depressive illness, political instabilities and high disease burdens including HIV/AIDS disproportionately affect children and adolescents in SSA [6, 11], and places them at a much higher risk of suicide, especially in those who fail to cope with these upheavals [12]. Moreover, SSA is home to the highest population of children and adolescents, with 20% of the world’s children and adolescents living in SSA. In South Africa, suicidality has been reported at suicide attempt 3.2%, suicide planning 5.8% and suicidal ideation 7.2% [11]. The few studies that have documented the burden and risk factors of suicide among children and adolescents in SSA have reported conflicting results, perhaps as a result of differences in the methods of assessment [13].

There is an urgent need to summarise the available literature about the burden and risk factors for suicide among children and adolescents in SSA. Findings from the synthesis of this data will be useful in developing specific evidence-based prevention and treatment strategies. Findings from this review will also be used in enlightening parents, children, adolescents and members of the community at large about the burden of suicide and the factors that predispose individuals to commit suicide.

For this review, we will (a) determine the overall prevalence of suicidal ideation, suicidal attempt and completed suicide among children and adolescents in SSA; (b) describe the methods used for suicidal attempt and completed suicide among children and adolescents in SSA; and (c) document the risk factors for suicidal ideation, suicidal attempt and completed suicide among children and adolescents in SSA.

## Methods

The review will be conducted and reported in accordance to the preferred reporting items for systematic reviews and meta-analysis (PRISMA) statement (Additional file 1) [14, 15]. This systematic review protocol title has been registered with the International Prospective Register of Systematic Reviews (PROSPERO) database (registration number: CRD42016048610).

### Eligibility criteria

We will include articles that have documented the prevalence and risk factors for suicidal ideation, suicidal attempt and completed suicide among children and adolescents aged 5–19 years in SSA. The review will include articles from studies done following cross-sectional, longitudinal, cohort and case control study designs. Sub-group analysis will be performed on the included studies to cater for the variations in the study designs.

Unpublished but accessible studies will be included in the review. Studies on non-suicidal deliberate self-harm will be excluded. Articles with only abstracts available (with no full articles) will be excluded as they may be difficult to compare with full articles.

The selection criteria of the articles for inclusion will be strictly applied and no double reporting of the same outcome will be included in the review. We will report the study as a single one, but in the event that there were separate outcomes, we will report them as such. Each publication will have the name of the author, year and labelled a, b, c, d.

### Study design

We will conduct a systematic review of observational studies (*cross*-*sectional*, *cohort*, *longitudinal* and *case control studies*) that documented the prevalence of completed suicide, suicide attempt or suicidal ideation among children and adolescents living in SSA irrespective of how the studies report the main outcome. We will also review suicide methods used and associated risk factors for suicide.

### Setting

The systematic review will only include studies conducted in sub-Saharan Africa.

### Participants

The participants in this systematic review will be children and adolescents aged 5–19 years living in SSA.

### Comparisons

The comparison groups will be children and adolescents without suicidal ideation, suicidal attempt and completed suicide.

### Outcomes

The outcomes of interest in this systematic review will be [1] prevalence, [2], suicidal methods used and [3] risk factors for suicidal ideation, attempts and completion among children and adolescents in Sub-Saharan Africa.

### Search methods

The search strategy will be carried out by the research team using the following electronic databases and search engines, from inception, using the same search strategy with alterations as appropriate for each database: the Cochrane Library, PsychINFO, PubMed, EMBASE, Africa wide-information and global health. We will hand search the references of the included studies. We will develop a Medline search strategy and use a combination of mesh terms, text words and combine them with appropriate Boolen operators in order to identify as many studies as possible. Grey literature will also been included. We will identify the relevant grey literature using web searching, web-based catalogues as well as using bibliographic databases.

#### Search string

We will search using various terms as shown in Appendix.

### Data extraction

The summary of the variables from which data will be extracted from reviewed articles include citation of the study, country/region where the study was done, year when data collection was carried out, study design, sample size, response rate, method of data collection, study population, age of study participants, selection criteria of the study participants, gender, religion, ethnicity, prevalence of suicidal ideation, prevalence of attempted suicide, prevalence of completed suicide, method of data analysis and sampling criteria used in participant selection. Quality of the study will be assessed through risk of bias assessment tool which will be done using a checklist developed from the strobe statement [16].

The data collected will also include the authors’ names, the title of the article and the year the study was conducted. This data will be extracted by two content experts on the research team. For articles with missing information, the primary authors will be contacted by the principle investigator to provide the missing or additional data. For any discrepancies, the principle investigator will make the final decision.

Data extraction will be done in two stages. Mr. Wilson Adriko, an information scientist and Dr. Elizabeth Kemigisha, a Paediatrician with interest in adolescent health will screen the title and abstract of all identified studies. We will then download the full text articles for further screening by two independent reviewers: Dr. Moses Ocan, a Pharmacologist with expertise in conducting reviews and Dr. Dickens H. Akena, a Psychiatrist and systematic review specialist. In the event that there is disagreement about which study to include or exclude, the PI who is a content expert will be the arbitrator.

### Quality assessment

Data will be entered in REVMAN 5.1.2 software for analysis of findings. The assessment of the methodological quality of the articles will be done using QUIPS (Quality in Prognostic Studies). We will assess study participation, confounding measurement and handling, outcome measurement and statistical analysis, and presentation.

The quality of primary studies will be ensured as part of the selection criteria of the articles prior to inclusion into the systematic review. This will be done by ensuring that only studies that meet the a priori selection criteria will be included in the review [17]. Screening of articles for inclusion into the review will be done independently by two reviewers. Articles will only be included if the Kappa agreement between the two reviewers in ≥ 70%. In addition, risk of bias (publication bias, methodological bias) in the included articles will be assessed using a risk of bias assessment checklist developed from the Cochrane risk of bias (RoB) assessment tool. We will use the AMSTAR [18] guidelines to assess for the quality of included studies, and GRADE [19] to assess the overall quality of each of the outcomes.

### Data synthesis

#### Qualitative synthesis

We will describe the important study features like date, number of participants, age categories, prevalence, completed suicide, attempted suicide, suicidal ideation, country in which the study was done and the associated risk factors. We will export the data to STATA 13.1 for analysis.

#### Meta-analysis

Statistical tests for heterogeneity will be used to assess the degree of variability in the prevalence measures between the included studies. Specifically, we will use the *I*^2^ statistic to report the percentage of variation across studies that is due to heterogeneity [20]. This is preferred because it does not depend on the number of studies reviewed. The *I*^2^ score of 0 (zero) will be considered no heterogeneity, whereas a score of more than zero but less than 25% will be taken as low heterogeneity. On the other hand, an *I*^2^ score of more than 25% will be considered as moderate-high heterogeneity. In case we find no statistically significant heterogeneity (*I*^2^ = zero), we will go ahead and conduct meta-analysis. Otherwise, in circumstances of statistically significant heterogeneity, we will not conduct meta-analysis [21].

Random-effects models will be employed [22]. This model is used when the researcher thinks or knows that the effect/main outcome varies widely in the population. We will use random effects model, because we are not confident that all the variables we will identify will be measured in the same way and with the same values [23–25].

The prevalence, odds ratios and confidence intervals of individual studies will be presented in forest plots and we will generate a summary prevalence and confidence levels. There will also be sensitivity and sub-group analyses to determine the influence of selected independent variables on the effect size (suicidality). We will use funnel plots to show small-study effects that will help point towards publication bias.

In addition to the prevalence, we will also pool effect estimates relating to risk factors. This will be achieved through the random effects meta-analysis. Pooling of results will be done for the prevalence of suicidal ideation, attempt and completed suicide. This will only be done if there is need to proceed to meta-analysis. We will conduct the following sub-group analyses: study designs (cross sectional studies, case control studies, clinical trials and cohort studies), region (s) where the studies were conducted, gender and participant age. This is to ensure that only studies that report similar effect measures are compared. In the event that there is too much heterogeneity, we will summarise the studies as narrative reviews.

The included articles will report study variables of interest to the review differently. The interconversion of the measurements of interest will therefore depend on how the articles have reported and available data to that effect. However, the interconversion will be manually done whenever there is need. The review will use odds ratios (OR) in reporting the risk factors for suicidal ideation, attempted suicide and completed suicide. Using Stata 13, we shall perform meta-regression on articles with high heterogeneity. The subgroup and meta-regression analyses for levels of heterogeneity will be conducted since heterogeneity may often be missed or undetected in the included studies. The possible causes of variability that will need to be considered include variation in participants from the different settings, outcomes of the different studies (suicidal ideation, attempt, behaviour), varying degrees of bias in the different studies and varying power of the different studies.

Heterogeneity will be inferred from the *I*^2^-statistic (*I*^2^ = 25% (small heterogeneity), *I*^2^ = 50% (moderate heterogeneity), *I*^2^ = 75% (large heterogeneity)) generated using Stata 13 software [26].

## Discussion

This protocol describes a systematic review of observational studies reporting completed suicide, suicide attempt and suicidal ideation among children adolescent in sub-Saharan Africa. According to our knowledge, no previous systematic review specifically addressed this topic. We will summarise the methods used and results of observational studies specifically looking at the prevalence and risk factors for completed suicide and suicidal behaviour in Sub-Saharan Africa. We anticipate facing a challenge of varying methodologies and use of different terms to mean the same thing. We also anticipate the challenge of stigma and underreporting of suicide-related deaths or morbidity. In addition, studies may have been conducted in various study populations like adolescents with HIV, or in war torn areas, those in school, with few studies looking at the general population. There may also be unpublished studies conducted on the same subject in the region that we may not have access to, but we shall include any available grey literature. We anticipate that once this review is complete and published, our findings will be of interest to adolescents with suicidal behaviour, their families and caregivers, clinicians and other healthcare professionals, scientists and policy makers.

### Additional file

Additional file 1: PRISMA-P 2015 Checklist. (DOCX 81 kb)

## Appendix

### Search string

We will search using the following terms in the order 1–3.

1. Suicide) OR Para-suicide) OR suicide awareness) OR suicide attempt) OR attempted suicide) OR suicide ideation) OR potential suicide) OR deliberate self-harm) OR uncompleted suicide) OR completed suicide) OR suicidality
2. Children) OR child) OR boy) OR girl) OR juvenile) OR minors) OR paediatric) OR Adolescence) OR Preadolescence) OR Puberty) OR Teenager) OR Teen) OR Young) OR Youth
3. Developing countries) OR low income countries) OR resource-limited) OR resource constrained) OR Africa) OR Angola) OR Benin) OR Botswana) OR Burkina Faso) OR Burundi) OR Cameroon) OR Cape Verde) OR Central African Republic) OR Chad) OR Comoros) OR Congo) OR Democratic Republic of Congo) OR Djibouti) OR Equatorial Guinea) OR Eritrea) OR Ethiopia) OR Gabon) OR Gambia) OR Ghana) OR Guinea) OR Guinea Bissau) OR Ivory Coast) OR Cote d’Ivoire) OR Jamahiriya) OR Jamahiryia) OR Kenya) OR Lesotho) OR Liberia) OR Madagascar) OR Malawi) OR Mali) OR Mauritania) OR Mauritius) OR Mayote) OR Mozambique) OR Mozambique) OR Namibia) OR Niger) OR Nigeria) OR Principe) OR Reunion) OR Rwanda) OR Sao Tome) OR Senegal) OR Seychelles) OR Sierra Leone) OR Somalia) OR South Africa) OR St Helena) OR Sudan) OR Swaziland) OR Tanzania) OR Togo) OR Tunisia) OR Uganda) OR Western Sahara) OR South Suda) OR Zambia) OR Zimbabwe 657,258

## Abbreviations

SA: Suicide attempt
SI: Suicide ideation
WHO: World Health Organisation
