# Supplementary material for: A systematic review of the risk factors for suicidal ideation, suicidal attempt and completed suicide among children and adolescents in sub-Saharan Africa between 1986 and 2018: protocol for a systematic review of observational studies
Source: Syst Rev. 2018 Dec 12;7:230. doi: 10.1186/s13643-018-0901-8 (PMC6292009; doi:10.1186/s13643-018-0901-8)
Supplement: Supplementary file 1 — PRISMA-P 2015 Checklist. (DOCX 81 kb) [file 13643_2018_901_MOESM1_ESM.docx]

**PRISMA-P 2015 Checklist**

# **This checklist has been adapted for use with systematic review protocol submissions to BioMed Central journals from Table 3 in Moher D et al**:**** Preferred reporting items for systematic review and meta-analysis protocols (PRISMA-P) 2015 statement. *Systematic Reviews* 2015 ****4****:1

# An Editorial from the Editors-in-Chief of *Systematic Reviews* details why this checklist was adapted - **Moher D, Stewart L & Shekelle P**:**** Implementing PRISMA-P: recommendations for prospective authors. *Systematic Reviews* 2016 ****5****:15

| **Section/topic** | **#** | **Checklist item** | **Information reported** | | **Line number(s)** |
| --- | --- | --- | --- | --- | --- |
|  |  |  | **Yes** | **No** |  |
| **ADMINISTRATIVE INFORMATION** | | | | | |
| **Title** | | | | | |
| Identification | 1a | A systematic review of the risk factors for suicide among children and adolescents in sub-Saharan Africa between 1986 and 2016: protocol for a systematic review of observational studies |  |  |  |
| Update | 1b | If the protocol is for an update of a previous systematic review, identify as such **N/A** |  |  |  |
| **Registration** | 2 | PROSPERO International prospective register of systematic reviews: CRD42016048610 |  |  |  |
| **Authors** | | | | | |
| Contact | 3a | Godfrey Zari Rukundo: [grukundo@must.ac.ug](mailto:grukundo@must.ac.ug) **(Corresponding Author)**  Mbarara-Kabale road,  Department of Psychiatry, Mbarara University of Science and Technology and African Centre for Suicide Prevention and Research  Elizabeth Kemigisha: [ekemigisha@must.ac.ug](mailto:ekemigisha@must.ac.ug)  Faculty of Interdisciplinary Studies, Mbarara University of Science and Technology  Moses Ocan: [ocanmoses@gmail.com](mailto:ocanmoses@gmail.com)  Department of Pharmacology & Therapeutics, Makerere College of Health Sciences  Wilson Adriko: wadriko@must.ac.ug  Library, Mbarara University of Science and Technology  Dickens Howard Akena: [akenadickens@yahoo.co.uk](mailto:akenadickens@yahoo.co.uk)  Department of Psychiatry, Makerere College of Health Sciences and Africa Centre for Systematic Reviews |  |  |  |
| Contributions | 3b | - Content expert/ guarantor of the review: Dr. G. Z. Rukundo, Psychiatrist with a PhD in suicidology - Search strategist: Mr. Wilson Adriko - Data abstraction and extraction and entry: Dr. Elizabeth Kemigisha, a Paediatrician, and Moses Ocan, a Pharmacologist. - Dr. Dickens Akena a Psychiatrist and systematic review specialist based at the African Centre for Systematic Reviews   All authors read and approved the final manuscript |  |  |  |
| **Amendments** | 4 | If the protocol represents an amendment of a previously completed or published protocol, identify as such and list changes; otherwise, state plan for documenting important protocol amendments **N/A** |  |  |  |
| **Support** | | | | | |
| Sources | 5a | No dedicated funding for this systematic review. |  |  |  |
| Sponsor | 5b | Provide name for the review funder and/or sponsor **N/A** |  |  |  |
| Role of sponsor/funder | 5c | Describe roles of funder(s), sponsor(s), and/or institution(s), if any, in developing the protocol **N/A** |  |  |  |
| **INTRODUCTION** | | | | | |
| **Rationale** | 6 | Suicide is one of the leading causes of death among children and adolescents. Most studies about the burden and risk factors for suicide have been conducted in high income countries. However, there is a dearth in the literature about the burden and risk factors for suicide among children and adolescents in low and middle income countries including within Sub-Saharan Africa (SSA). There is an urgent need to summarize the available literature about the burden and risk factors for suicide among children and adolescents in SSA. |  |  |  |
| **Objectives** | 7 | In this review, we will  a) Determine the overall prevalence of suicidal ideation, suicidal attempt, and completed suicide among children and adolescents in SSA,  b) describe the methods used to assess suicidal ideation, suicidal attempt, and completed suicide among children and adolescents in SSA, and  c) Document the risk factors for suicidal ideation, suicidal attempt, and completed suicide among children and adolescents in SSA. |  |  |  |
| **METHODS** | | | | | |
| **Eligibility criteria** | 8 | We will conclude journal articles that have documented the prevalence and risk factors for suicidal ideation, suicidal attempt and completed suicide among children and adolescents aged 5-19 years in SSA. We will exclude articles of other study designs (not observational) and studies conducted in other parts of the world will be excluded. Unpublished studies will be excluded since only journal articles will be included in the review. Qualitative studies will be excluded from the study since they are limited in estimating prevalence. Studies on non-suicidal deliberate self-harm will be excluded. Articles with only abstracts available (with no full articles) will be excluded as they may be difficult to compare with full articles. |  |  |  |
| **Information sources** | 9 | The search strategy will be carried out by the research team using the following electronic databases and search engines, from inception, using the same search strategy with alterations as appropriate for each database: the Cochrane Library, PsychINFO, PubMed, EMBASE, Africa wide-information and global health. We will hand search the references of the included studies. |  |  |  |
| **Search strategy** | 10 | We will search data spanning 1986-2016 using the following terms in the order 1-3.   1. Suicide) OR Para-suicide) OR suicide awareness) OR suicide attempt) OR attempted suicide) OR suicide ideation) OR potential suicide) OR deliberate self harm) OR uncompleted suicide) OR completed suicide) OR suicidality 2. Children) OR child) OR boy) OR girl) OR juvenile) OR minors) OR paediatric) OR Adolescence) OR Preadolescence) OR Puberty) OR Teenager) OR Teen) OR Young) OR Youth   Developing countries) OR low income countries) OR resource-limited) OR resource constrained) OR Africa) OR Angola) OR Benin) OR Botswana) OR Burkina Faso) OR Burundi) OR Cameroon) OR Cape Verde) OR Central African Republic) OR Chad) OR Comoros) OR Congo) OR Democratic Republic of Congo) OR Djibouti) OR Equatorial Guinea) OR Eritrea) OR Ethiopia) OR Gabon) OR Gambia) OR Ghana) OR Guinea) OR Guinea Bissau) OR Ivory Coast) OR Cote d’Ivoire) OR Jamahiriya) OR Jamahiryia) OR Kenya) OR Lesotho) OR Liberia) OR Madagascar) OR Malawi) OR Mali) OR Mauritania) OR Mauritius) OR Mayote) OR Mozambique) OR Mozambique) OR Namibia) OR Niger) OR Nigeria) OR Principe) OR Reunion) OR Rwanda) OR Sao Tome) OR Senegal) OR Seychelles) OR Sierra Leone) OR Somalia) OR South Africa) OR St Helena) OR Sudan) OR Swaziland) OR Tanzania) OR Togo) OR Tunisia) OR Uganda) OR Western Sahara) OR South Suda) OR Zambia) OR Zimbabwe 657258 |  |  |  |
| ***STUDY RECORDS*** | | | | | |
| Data management | 11a | Endnote citation manager will be used and articles will be downloaded. An Endnote library will be created specifically for this systematic review. |  |  |  |
| Selection process | 11b | All article titles will be reviewed in order to exclude non-eligible ones. This review will be limited to publications done between 1986 and 2016. |  |  |  |
| Data collection process | 11c | The key data that will be collected include prevalence of completed suicide, suicidal attempt, suicidal ideation, most common risk factors associated with suicide, socio-demographics (age, gender, religion, ethnicity, etc) and country in which the study was conducted. The data collected will also include the authors’ names, the title of the article, study design and the year the study was conducted. This data will be extracted by two content experts on the research team. For articles with missing information, the primary authors will be contacted by the principle investigator to provide the missing or additional data. For any discrepancies, the principle investigator will make the final decision.  Data extraction will be done in two stages. Mr. Wilson Adriko, an information scientist and Dr. Elizabeth Kemigisha, a Paediatrician with interest in adolescent health will screen the title and abstract of all identified studies. We will then download the full text articles for further screening by two independent reviewers: Dr. Moses Ocan, a Pharmacologist with expertise in conducting reviews and Dr. Dickens H. Akena, a Psychiatrist and systematic review specialist. In the event that there is disagreement about which study to include or exclude, the PI who is a content expert will be the arbitrator. |  |  |  |
| **Data items** | 12 | We will conduct a systematic review of observational studies that documented the prevalence of completed suicide, suicide attempt or suicidal ideation among children and adolescents living in SSA.  The participants in this systematic review will children and adolescents aged 5-19 years living in SSA.  The comparison groups will be children and adolescents without Suicidal ideation, suicidal attempt and completed suicide. |  |  |  |
| **Outcomes and prioritization** | 13 | The outcomes of interest in this systematic review will be Suicidal ideation, suicidal attempt and completed suicide, as well as the associated risk factors among children and adolescents in sub-Saharan Africa. |  |  |  |
| **Risk of bias in individual studies** | 14 | The risk of bias of the articles will be assessed using Cochrane risk of bias (RoB) assessment tool for non-randomized studies. In addition, the GRADE will be utilized to establish strength of recommendations and level of confidence in the results of meta-analyses reported in the different studies |  |  |  |
| ***DATA*** | | | | | |
| **Synthesis** | 15a | Statistical tests for heterogeneity will be used to assess the degree of variability in the prevalence measures between the included studies. Random-effects models will be employed. The prevalence, odds ratios and confidence intervals of individual studies will be presented in forest plots and we will generate a summary prevalence and confidence levels. There will also be sensitivity and sub-group analyses to determine the influence of selected independent variables on the effect size (suicidality).  Publication bias will be assessed using funnel plots. |  |  |  |
|  | 15b | The prevalence, odds ratios and confidence intervals of individual studies will be presented in forest plots and we will generate a summary prevalence and confidence levels. |  |  |  |
|  | 15c | There will also be sensitivity and sub-group analyses to determine the influence of selected independent variables on the effect size (suicidality). |  |  |  |
|  | 15d | During qualitative synthesis, we will describe the important study features like date, number of participants, age categories, prevalence, completed suicide, attempted suicide, suicidal ideation, country in which the study was done and the associated risk factors. We will export the data to STATA 13.1 for analysis. |  |  |  |
| **Meta-bias(es)** | 16 | Publication bias will be assessed using funnel plots. |  |  |  |
| **Confidence in cumulative evidence** | 17 | Data will be entered in REVMAN 5.1.2 software for analysis of findings. The assessment of the methodological quality of the articles will be done using QUIPS (Quality in Prognostic Studies). |  |  |  |
